# Molecular expression profiling with respect to KEGG hsa05219 pathway

**DOI:** 10.3332/ecancer.2011.189

**Published:** 2011-02-22

**Authors:** Raghavendra Krishnappa

**Affiliations:** Life Science/Healthcare Vertical, MphasiS Limited, Chennai, India

## Abstract

One of the most promising avenues for interpreting large datasets of molecular expression profiles involves pathway-based analysis. Pathways are collection of genes and proteins that perform a well-defined biological task. These pathways have been established through decades of molecular biology research and are collected in a variety of public pathway repositories (KEGG and Reactome Pathway database). Understanding the complexity of these pathways is critical for understanding normal biological conditions and disease states and also since the number of known pathways within the cells is significantly smaller than the number of genes that is typically profiled, the transformation of data from a gene-centric view to a pathway-centred one represents a dramatic reduction in the number of dimensions. Such reduction allows a biologist to interpret and understand the data in a manner that is not possible when it is viewed as a collection of individual genes.

## Introduction

Gene expression studies are used as an independent predictive method for prognosis. In cancer genomic studies, tremendous effort has been devoted to pathway-based analysis. Pathway analysis is a promising tool to identify the mechanisms that underlie disease, adaptive physiological compensatory responses and new avenues for investigation. Different pathways have different biological functions. Thus, it is reasonable to study each pathway separately. Among the many pathways, only a few have predictive power for cancer development. Among genes within predictive pathways, there are subsets having small to moderate predictive power, whereas the remaining are noisy genes [1–6].

## Background

Genes have the inherent pathway structure, where pathways are composed of multiple genes with coordinated functions. The aim of this study was to identify genetic signatures associated with disease prognosis in bladder cancer with respect to hsa05219 pathway obtained from Kyoto Encyclopedia of Genes and Genomes (KEGG).

## Methods

Microarray data files were taken from Gene Expression Omnibus (GEO), accession number GSE7476. Four different types of data files were generated from GSE7476 experiment by analyzing gene expression profiles in normal bladder tissues (controls), low grade superficial tumour samples (pathologically classified as Ta low grade, named as Ta), high grade superficial tumours with an unclear clinical behaviour (T1 high grade, named as T1) and high grade muscle invasive tumours (pathologically classified as T2, T3 or T4, named as T2+).

Data files representing controls and high grade invasive tumours (T2+) were compared for the current review work.

## Affymetrix data files

Affymetrix gene expression chip was used for their study and the intensity values seen in the data file were log transformed values. One would subtract the control value from the experimental value to find the significant change in expression level. ‘Researchers generally’ use a cut off at least 2-fold change (linear value) between control and experiment to ‘screen significantly differentially expressed genes’.

Affymetrix does not have a certain threshold cut off that it recommends. Researchers in the community have seemed to adopt a value of about 100 (linear) so ∼6.65 (log). However, this does not mean that a value of 6.8 is expressed in a sample; this is just a general guideline that many researchers have adopted to filter out a bulk of the probe sets. Most researchers will first analyze the data based on fold change, then filter on intensity when a probe set is <100 in both samples. A change from 3.5 to 4.8 in a sample could very well be just a background, where as a change from 6.0 to 7.4 might be real.

## hsa05219 pathway

Pathway hsa05219 referring to bladder cancer was selected from KEGG pathway database (section 6.1 cancers http://www.genome.jp/kegg/pathway.html). There are totally 42 genes listed in hsa05219 pathway which are believed to be involved in causing bladder cancer (Tables 1–3).

## Conclusion

We have taken the list of genes associated with bladder cancer pathway from KEGG database. Log difference between the control and study subjects which exceeds more than 1.0 or less than −1.0 were first screened. *THBS1*, *RPS6KA5* and *CDKN1A* are the genes which are highly expressed in control when compared with study subjects (T2+). These genes are associated with ‘angiogenesis’, ‘mitogen-activated protein kinase (MAPK) signaling pathway’ and ‘cell cycle’, respectively. *ERBB2*, *TYMP*, *CDH1*, *TP53*, *DAPK1*, *CCND1*, *FGFR3*, *KRAS*, *E2F3*, *CDKN2A*, *VEGFA*, *MMP1* are the genes which are highly expressed in study when compared to control and these genes are associated with ‘ErbB signaling pathway’, ‘nucleotide metabolism’ ‘adherens junction’, ‘p53 pathway’, ‘cell cycle’, ‘MAPK signaling pathway’ and ‘angiogenesis’. By this current pathway analysis approach to the GSE7476 bladder cancer datasets, we can say that genes like *ERBB2*, *TYMP*, *CDH1*, *TP53*, *DAPK1*, *CCND1*, *FGFR3*, *KRAS*, *E2F3*, *CDKN2A*, *VEGFA*, *MMP1* can be used as prognosis markers for bladder cancer gene expression study. Association of above 12 sets of genes for causing cancer was confirmed from Online Mendelian Inheritance in Man (OMIM) and articles from PubMed database. Further research is needed to evaluate whether the same gene signatures result from other bladder cancer profiling experiments (Table 4).

## Difference in expression level

### Significant findings

According to KEGG pathway, ‘*hsa05219*’ for bladder cancer, *RB*, *CDKN2A* and *p53* are considered as tumour suppressor genes and *FGFR3* and *HRAS* as oncogenes. Two of the tumour suppressor genes *CDKN2A* and *p53* were expressed in significant level when compared with normal tissue samples. *FGFR3* which is an oncogene is highly expressed in tumour samples compared to control tissue samples. *CDKN2A*, *p53* and *FGFR3* along with the other genes *ERBB2*, *TYMP*, *CDH1*, *DAPK1*, *CCND1*, *KRAS*, *E2F3*, *VEGFA* and *MMP1* are unregulated in tumour tissue samples. All these genes play an important role in ErbB signaling pathway, nucleotide metabolism, adherens junction, p53 pathway, MAPK signaling pathway, cell cycle and angiogenesis. *THBS1*, *RPS6KA5* and *CDKN1A* are downregulated in tumour tissue when compared with control tissue samples. Out of the 42 genes listed in the KEGG bladder cancer pathway, only eight genes *RASSF1*, *RB1*, *HRAS*, *EGFR*, *ERBB2*, *DAPK1*, *FGFR3* and *CDKN2A* have reference support to prove their involvement in causing bladder cancer. This current review suggest the lack of research/involvement of the other genes in the pathway to cause bladder cancer. Pathway analysis of affymetrix data file shows upregulation of four genes *ERBB2*, *DAPK1*, *FGFR3* and *CDKN2A* which have reference to prove their involvement in causing bladder cancer.

## Figures and Tables

**Table 1:** Gene list from hsa05219 pathway showing affymetrix ID, gene name, and gene ID

**Sl no.**	**Affy ID**	**Gene name**	**Gene ID**
1	201109_s_at	*THBS1*	7057
2	204633_s_at	*RPS6KA5*	9252
3	202284_s_at	*CDKN1A*	1026
4	209946_at	*VEGFC*	7424
5	202431_s_at	*MYC*	4609
6	204346_s_at	*RASSF1*	11186
7	203683_s_at	*VEGFB*	7423
8	224621_at	*MAPK1*	5594
9	206742_at	*FIGF*	2277
10	243829_at	*BRAF*	673
11	212046_x_at	*MAPK3*	5595
12	206324_s_at	*DAPK2*	23604
13	215179_x_at	*PGF*	5228
14	1566678_at	*MMP2*	4313
15	206254_at	*EGF*	1950
16	203132_at	*RB1*	5925
17	212983_at	*HRAS*	3265
18	201895_at	*ARAF*	369
19	202424_at	*MAP2K2*	5605
20	203891_s_at	*DAPK3*	1613
21	211506_s_at	*IL8*	3576
22	201244_s_at	*RAF1*	5894
23	202670_at	*MAP2K1*	5604
24	204947_at	*E2F1*	1869
25	203936_s_at	*MMP9*	4318
26	211607_x_at	*EGFR*	1956
27	228361_at	*E2F2*	1870
28	202246_s_at	*CDK4*	1019
29	202647_s_at	*NRAS*	4893
30	225160_x_at	*MDM2*	4193
31	216836_s_at	*ERBB2*	2064
32	204858_s_at	*TYMP*	1890
33	201131_s_at	*CDH1*	999
34	211300_s_at	*TP53*	7157
35	203139_at	*DAPK1*	1612
36	208712_at	*CCND1*	595
37	204379_s_at	*FGFR3*	2261
38	214352_s_at	*KRAS*	3845
39	203693_s_at	*E2F3*	1871
40	209644_x_at	*CDKN2A*	1029
41	211527_x_at	*VEGFA*	7422
42	204475_at	*MMP1*	4312

**Table 2:** Displaying affymetrix grades and ID along with T2+ and control mean values

**Sl no.**	**Affy grades**	**Affy ID**	**T2+ average**	**C average**	**Difference**
1	A	201109_s_at	7.197941667	9.268797667	−2.070856
2	A	204633_s_at	5.538698	6.974227667	−1.435529667
3	A	202284_s_at	8.292146333	9.426557667	−1.134411333
4	A	209946_at	4.972374667	5.932246333	−0.959871667
5	A	202431_s_at	9.570811333	10.20563933	−0.634828
6	A	204346_s_at	5.68049	6.300829667	−0.620339667
7	A	203683_s_at	4.670015333	5.256459	−0.586443667
8	A	224621_at	8.188972333	8.710883	−0.521910667
9	A	206742_at	3.331917667	3.622964	−0.291046333
10	A	243829_at	5.140807	5.374049333	−0.233242333
11	A	212046_x_at	7.135151667	7.303775667	−0.168624
12	A	206324_s_at	4.684244667	4.824944667	− 0.1407
13	A	215179_x_at	7.105821667	7.123966	− 0.018144333
14	A	1566678_at	3.752619667	3.757442667	−0.004823
15	A	206254_at	3.159667333	3.112292333	0.047375
16	A	203132_at	6.140205333	6.082435667	0.057769667
17	A	212983_at	6.390220333	6.299536	0.090684333
18	A	201895_at	7.477000333	7.315159	0.161841333
19	A	202424_at	8.049549333	7.880261667	0.169287667
20	A	203891_s_at	6.282708	6.019414667	0.263293333
21	A	211506_s_at	7.225747667	6.952306667	0.273441
22	A	201244_s_at	8.239435333	7.863649667	0.375785667
23	A	202670_at	7.602015	7.090296333	0.511718667
24	A	204947_at	5.903176333	5.286578333	0.616598
25	A	203936_s_at	6.152433667	5.477768667	0.674665
26	A	211607_x_at	5.508033333	4.786028667	0.722004667
27	A	228361_at	5.898566667	5.131460667	0.767106
28	A	202246_s_at	8.944539333	8.051693	0.892846333
29	A	202647_s_at	6.692538	5.796430333	0.896107667
30	A	225160_x_at	7.044023333	6.121776667	0.922246667
31	A	216836_s_at	9.437352333	8.389638667	1.047713667
32	A	204858_s_at	6.671381333	5.556419	1.114962333
33	A	201131_s_at	10.37200433	9.242416333	1.129588
34	A	211300_s_at	5.945064333	4.792846667	1.152217667
35	A	203139_at	8.916442667	7.673051333	1.243391333
36	A	208712_at	8.620222333	7.024218667	1.596003667
37	A	204379_s_at	9.858403	8.250372333	1.608030667
38	A	214352_s_at	9.427273667	7.638116667	1.789157
39	A	203693_s_at	7.203678333	5.31342	1.890258333
40	A	209644_x_at	7.779116333	5.662257333	2.116859
41	A	211527_x_at	8.164427333	6.013361333	2.151066
42	A	204475_at	8.622475667	4.105322667	4.517153

Log difference between the control and study subjects which exceeds more than 1.0 or less than −1.0 were first screened. Downregulated genes are marked in green and upregulated genes are marked in red with respect to tumour samples.

**Table 3:** List displaying up and downregulated genes, downregulated genes are marked in green colour and up regulated genes are marked in red colour with respect to tumour samples

**Sl No**	**Gene name**	**Difference**	**Pathways involved**
1	*THBS1*	−2.07086	Angiogenesis
2	*RPS6KA5*	−1.43553	MAPK signaling pathway
3	*CDKN1A*	−1.13441	Cell cycle
4	*ERBB2*	1.047714	ErbB signaling pathway
5	*TYMP*	1.114962	Nucleotide metabolism
6	*CDH1*	1.129588	Adherens junction
7	*TP53*	1.152218	p53 pathway—tumour suppressor
8	*DAPK1*	1.243391	MAPK signaling pathway
9	*CCND1*	1.596004	Cell cycle
10	*FGFR3*	1.608031	MAPK signaling pathway
11	*KRAS*	1.789157	MAPK signaling pathway
12	*E2F3*	1.890258	Cell cycle
13	*CDKN2A*	2.116859	Cell cycle—tumour suppressor
14	*VEGFA*	2.151066	Angiogenesis
15	*MMP1*	4.517153	Angiogenesis

MAPK, mitogen-activated protein kinase.

**Table 4:** Genes from the hsa05219 pathway involved in different cancers

**Gene name**	**Diff (N-T2+)**	**From reference article**
*THBS1*	−2.070856	Not related to any cancer
*RPS6KA5*	−1.435529667	Not related to any cancer
*CDKN1A*	−1.134411333	Cervical cancer
*VEGFC*	−0.959871667	Gastric cancer
*MYC*	−0.634828	Acute lymphoblastic leukaemia (ALL) (precursor B lymphoblastic leukaemia), ALL (precursor T lymphoblastic leukaemia), Burkitt lymphoma, multiple myeloma, small cell lung cancer, oral cancer, penile cancer, ovarian cancer, choriocarcinoma, breast cancer, osteosarcoma, Kaposi’s sarcoma, laryngeal cancer
*RASSF1*	−0.620339667	Non-small cell lung cancer, bladder cancer, nasopharyngeal cancer
*VEGFB*	−0.586443667	Gastric cancer
*MAPK1*	−0.521910667	Not related to any cancer
*FIGF*	−0.291046333	Gastric cancer
*BRAF*	−0.233242333	Thyroid and malignant cancer
*MAPK3*	−0.168624	Not related to any cancer
*DAPK2*	−0.1407	Not related to any cancer
*PGF*	−0.018144333	Not related to any cancer
*MMP2*	−0.004823	Choriocarcinoma
*EGF*	0.047375	Gastric cancer
*RB1*	0.057769667	Chronic myeloid leukaemia (CML), small cell lung cancer, oesophageal cancer, breast cancer, osteosarcoma, glioma, hepatocellular carcinoma
*HRAS*	0.090684333	Bladder, penile, cervical, thyroid cancer, squamous cell carcinoma, hepatocellular carcinoma
*ARAF*	0.161841333	Not related to any cancer
*MAP2K2*	0.169287667	Not related to any cancer
*DAPK3*	0.263293333	Not related to any cancer
*IL8*	0.273441	Not related to any cancer
*RAF1*	0.375785667	Not related to any cancer
*MAP2K1*	0.511718667	Not related to any cancer
*E2F1*	0.616598	Not related to any cancer
*MMP9*	0.674665	Penile cancer
*EGFR*	0.722004667	Oral cancer, oesophageal, gastric, bladder, cervical, laryngeal cancer, glioma and choriocarcinoma
*E2F2*	0.767106	Not related to any cancer
*CDK4*	0.892846333	Cervical cancer, malignant melanoma, glioma
*NRAS*	0.896107667	Acute myeloid leukaemia (AML), multiple myeloma, oral cancer, thyroid cancer, adrenal carcinoma, malignant melanoma, hepatocellular carcinoma, autoimmune lymphoproliferative syndromes
*MDM2*	0.922246667	Penile cancer, choriocarcinoma, osteosarcoma, alveolar rhabdmycosarcoma and glioma
*ERBB2*	1.047713667	Gastric, pancreatic, bladder, endometrial, ovarian, cervical, breast cancer, choriocarcinoma, cholangiocarcinoma
*TYMP*	1.114962333	Not related to any cancer
*CDH1*	1.129588	Gastric, penile, breast, thyroid, nasopharyngeal cancer and hepatocellular carcinoma
*TP53*	1.152217667	MAPK signaling pathway, cell cycle, p53 signaling pathway, apoptosis, Wnt signaling pathway, neurotrophin signaling pathway, amyotrophic lateral sclerosis, Huntington’s disease, pathways in cancer, colorectal cancer, pancreatic cancer, endometrial cancer, glioma, prostate cancer, thyroid cancer, basal cell carcinoma, melanoma, bladder cancer, CML, small cell lung cancer, non-small cell lung cancer
*DAPK1*	1.243391333	Bladder cancer
*CCND1*	1.596003667	Hairy-cell leukemia, multiple myeloma, oral cancer, oesophageal cancer, breast cancer, laryngeal cancer
*FGFR3*	1.608030667	Multiple myeloma and bladder cancer
*KRAS*	1.789157	AML, multiple myeloma, non-small cell lung cancer, oral cancer, gastric cancer, pancreatic cancer, colorectal cancer, endometrial cancer, ovarian cancer, cervical cancer, thyroid cancer, squamous cell carcinoma, Kaposi’s sarcoma, cholangiocarcinoma, gallbladder cancer, hepatocellular carcinoma
*E2F3*	1.890258333	Not related to any cancer
*CDKN2A*	2.116859	CML, Burkitt lymphoma, adult T-cell leukemia, non-small cell lung cancer, malignant pleural mesothelioma, oral cancer, oesophageal cancer, pancreatic cancer, bladder cancer, penile cancer, osteosarcoma, malignant melanoma, squamous cell carcinoma, glioma, malignant islet cell carcinoma, cholangiocarcinoma, gallbladder cancer, hepatocellular carcinoma, nasopharyngeal cancer, laryngeal cancer, type II diabetes mellitus
*VEGFA*	2.151066	Gastric cancer
*MMP1*	4.517153	Choriocarcinoma

Log difference between the control and study subjects which exceeds more than 1.0 or less than −1.0 were first screened. Downregulated genes are marked in green and upregulated genes are marked in red with respect to tumour samples.
